# Priority conservation area of *Larix gmelinii* under climate change: application of an ensemble modeling

**DOI:** 10.3389/fpls.2023.1177307

**Published:** 2023-05-09

**Authors:** Minglong Gao, Guanghua Zhao, Shuning Zhang, Zirui Wang, Xuanye Wen, Lei Liu, Chen Zhang, Niu Tie, Rula Sa

**Affiliations:** ^1^ College of Forestry, Inner Mongolia Agricultural University, Hohhot, China; ^2^ College of Life Sciences, Shanxi Normal University, Taiyuan, China; ^3^ Administrative office, Shanwei Middle School, Shanwei, China; ^4^ College of Forestry, Fujian Agriculture and Forestry University, Fuzhou, China; ^5^ Forestry and Grassland Bureau of Inner Mongolia Autonomous Region, Hohhot, China

**Keywords:** *Larix gmelinii*, biomod2, Marxan, climate change, potential distribution, conservation planning, niche changes

## Abstract

*Larix gmelinii* (Rupr.) Kuzen is a major tree species with high economic and ecological value in the Greater Khingan Mountains coniferous forest of Northeast China. Reconstructing the priority Conservation Area of *Larix gmelinii* under Climate could provide a scientific basis for its germplasm conservation and management. The present study used ensemble and Marxan model simulations to predict species distribution areas and delineate priority conservation areas for *Larix gmelinii* in relation to productivity characteristics, understory plant diversity characteristics, and climate change impacts. The study revealed that the Greater Khingan Mountains and the Xiaoxing'an Mountains, with an area of approximately 300 974.2 km^2^, were the most suitable for *L. gmelinii*. The stand productivity of *L. gmelinii* in the most suitable area was significantly higher than that in the less suitable and marginally suitable areas, but understory plant diversity was not dominant. The increase in temperature under future climate change scenarios will reduce the potential distribution and area under *L. gmelinii*; the species will migrate to higher latitudes of the Greater Khingan Mountains, while the degree of niche migration will gradually increase. Under the 2090s-SSP585 climate scenario, the most suitable area for *L. gmelinii* will completely disappear, and the climate model niche will be completely separated. Therefore, the protected area of *L. gmelinii* was demarcated with a target of the productivity characteristics, understory plant diversity characteristics and climate change sensitive area, and the current key protected area was 8.38 × 10^4^ km^2^. Overall, the study’s findings will lay a foundation for the protection and rational development and utilization of cold temperate coniferous forests dominated by *L. gmelinii* in the northern forested region of the Greater Khingan Mountains.

## 1 Introduction

Global warming caused by the annual increase in greenhouse gas emissions has become a global problem (Chamorro et al., 2020). The major abiotic factor affecting species distribution is climate, and biome changes may occur on nearly 35% of the Earth’s land surface if the global temperature increases by 4°C (Bezeng et al., 2017; Trindade et al., 2020; Villén-Peréz et al., 2020; IPCC, 2021). *Larix gmelinii* (Rupr.) Kuzen is a robust light-loving species with high water requirements and is widely distributed in the Greater Khingan Mountains (>70% area). It is considered a zonal vegetation of this area (Fu et al., 1999; Cui et al., 2000). Forests with *L. gmelinii* as the major tree species play a crucial role in air purification and water conservation in the Greater Khingan Mountains. Its wood also has high economic value and can be used for housing construction, civil engineering, electric poles, boats, joinery, and wood fiber industrial raw materials (Fu et al., 1999). Additionally, the content of arabinogalactan (AG) in the heartwood of *L. gmelinii* is the most abundant component, reaching approximately 30%. The species has been used in food and healthcare products (Grabner et al., 2005; Ito et al., 2020). An analysis of the tree rings showed that *L. gmelinii* is extremely sensitive to climate change, showing obvious changes in annual ring width (Sano et al., 2009). *L. gmelinii* forests with *L. gmelinii* as the main species often form a large area of simple forest. This species comprises China’s largest cold temperate forest, an ecosystem susceptible to global warming (Barchenkov, 2011).

With the development of geographic information technology, ecological models, and climate system models, species distribution models (SDMs) have gradually matured (Elith and Leathwick, 2009; Wiens et al., 2009). An SDM is typically based on the environment and the species’ living habits. By combining the data on species distribution with the environmental information of the corresponding location, an SDM predicts the spatial distribution pattern under the present and future climatic conditions and simulates the impact of the climate on species. As important tools for change response, SDMs have been widely used in research on species invasion, protection of endangered animals and plants, biological distribution in paleoclimate periods, and variations in species distribution under changing climatic conditions (Tanaka et al., 2020; Sun et al., 2021b; Wen et al., 2022). Similar to other ecological models, niche models exhibit prediction uncertainties, and these models are associated with model algorithms and parameters, species’ actual locations, and environmental variables; thus, selecting the most suitable model to estimate the suitable distribution range of different species is challenging (Pearson et al., 2006). Currently, researchers use random forest, the maximum entropy model, the generalized linear model, and the generalized additive model (Duque-Lazo et al., 2016; Arabameri et al., 2020). These SDMs employ different model algorithms and database schemes to determine the ecological dimensions and potential distributions based on different theoretical foundations and assumptions. Each model possesses unique advantages and disadvantages; however, none perfectly simulates species’ potential geographic distribution (Araújo and New, 2007).

The platform Biomod2, based on R software, provides ten commonly used SDM algorithms, and users can freely combine and customize the ensemble model for the studied species (Thuiller et al., 2009; Duque-Lazo et al., 2018). Although the inherent defects of each model cannot be avoided, assigning each model’s weight based on the TSS (true skill statistic) or ROC (receiver operating characteristic curve) will help achieve the best simulation effect in the ensemble model (Shabani et al., 2016; Hao et al., 2019).

Thus, the present study employed Biomod2 to analyze the appropriate/suitable distribution areas of *L. gmelinii* under different climatic conditions and the correlation between the stand structure and growth characteristics of natural *L. gmelinii* forests and the environmental variables. The study aimed to (1) predict the variations in *L. gmelinii* suitable areas under different climate scenarios, (2) analyze the crucial environmental features affecting the distribution of *L. gmelinii*, (3) analyze the changes in the *L. gmelinii* ecological niche under different future climatic conditions, (4) analyze the influence of habitat suitability on *L. gmelinii* stand productivity and understory biodiversity, and (5) zone the scope of *L. gmelinii* key protected areas. The study’s findings will lay a foundation for the protection and rational development and utilization of cold temperate coniferous forests dominated by *L. gmelinii* in the northern forests of the Greater Khingan Mountains.

## 2 Materials and methods

### 2.1 Collection of sample and species distribution records

**Figure 1:**
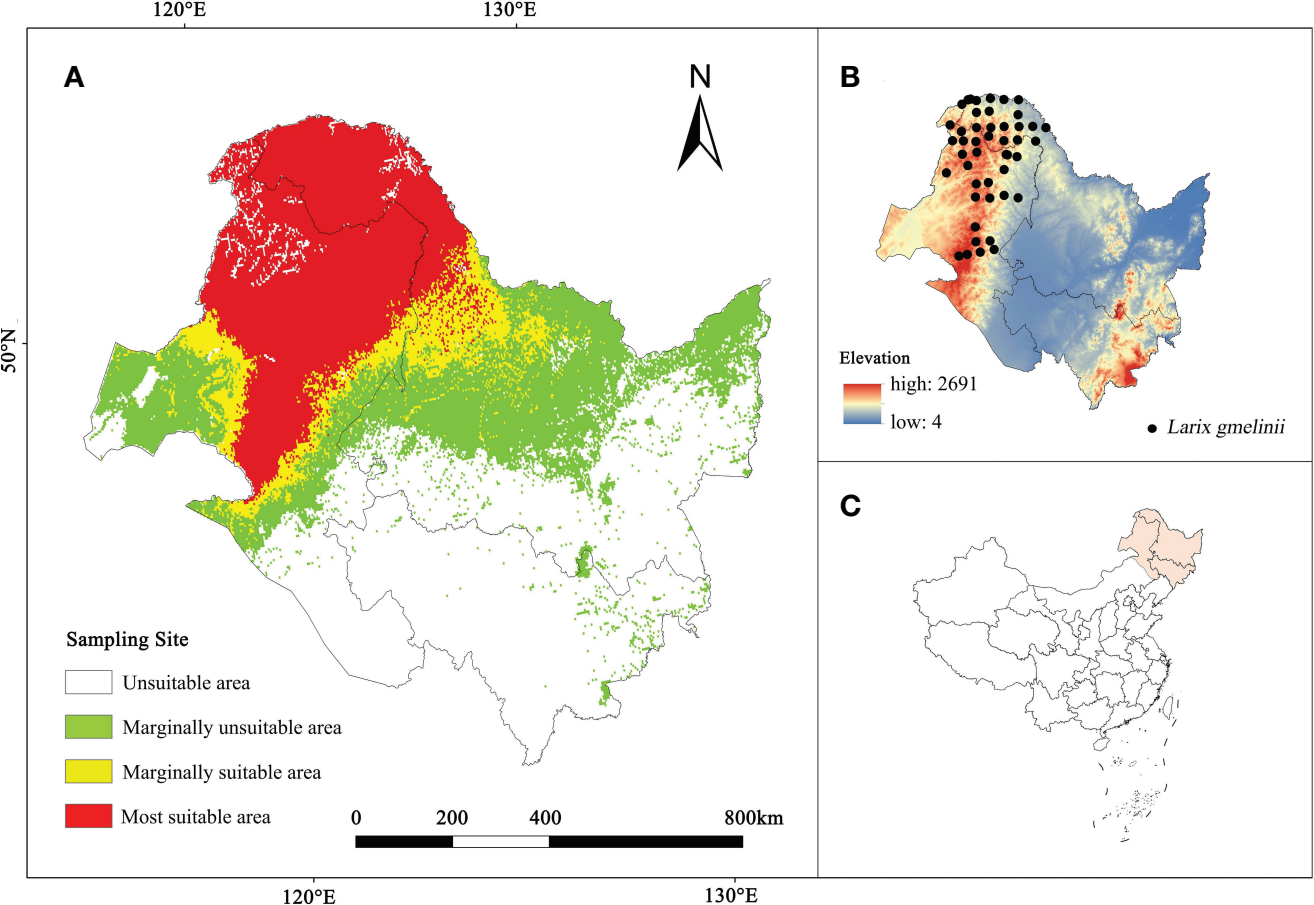
**(A)** The current habitat suitability area of *L. gmelinii*. **(B)** *L. gmelinii* sampling sites in China. **(C)** Location of the study area in China.

From 2017 to 2021, a total of 75 *L. gmelinii* distribution points were obtained through field investigations in the Inner Mongolia Autonomous Region and Heilongjiang Province. One distribution site (5 km × 5 km) was maintained per grid to avoid model overfitting caused by an excessive overconcentration of distribution sites. Finally, 46 valid samples were acquired, as shown in **Figures 1B, C**.

### 2.2 Selection and processing of environmental features for modeling

The study used 76 environmental features, including 54 climate factors, 14 soil factors, 4 terrain factors, 1 land cover factor, 1 human footprint factor, 1 normalized difference vegetation index (NDVI) factor, and 1 total primary productivity factor. We collected the current and future climate data from the WorldClim database (http://worldclim.org/data/index.html) (Fick and Hijmans, 2017). Here, future climate data under three shared socioeconomic pathway (SSP) scenarios were collected from the Beijing Climate Center Climate System Model (BCC-CSM2-MR), representing low (SSP126), medium (SSP245), and high (SSP585) concentrations of greenhouse gas emissions (Sun et al., 2021a). In addition, we collected soil and topographic data from the Harmonized World Soil Database (HWSD) of the Food and Agriculture Organization (http://www.fao.org/faostat/en/#data) (Milovac et al., 2018), human footprint data from the Human Footprint dataset (2009) in NASA’s Earth Observing System Data and Information System (EOSDIS) (Venter et al., 2016), land cover data from Tsinghua University’s Land Cover Remote Sensing Monitoring Raster Database (2017) (http://data.ess.tsinghua.edu.cn/fromglc10_2017v01.html), and NDVI data and total primary productivity data from the MODIS (https://ladsweb.modaps.eosdis.nasa.gov/search/order/1/MOD17A3) dataset. We obtained data on all variables at a 2.5 arc-minute spatial resolution (approximately 25 km).

To avoid the influence of multicollinearity of variables on the prediction accuracy (Yang et al., 2013), multicollinearity (Spearman correlation) and variance inflation factor (VIF) tests were performed for all environmental features in R (version 4.1.3). After eliminating the environmental factors with Spearman values< 0.7 and VIF< 5 (Elith et al., 2006; Zhao et al., 2021), nine climate variables (annual mean temperature, annual precipitation, isothermality, maximum temperature in the hottest month, mean temperature of the warmest quarter, minimum temperature in the coldest month, mean temperature of the coldest quarter, precipitation in September, and solar radiation in September), three soil variables (topsoil saturation, bulk density, and gravel content), two environmental variables (NDVI, primary productivity, and land cover), one human variable (footprint), and two topographic variables (elevation, landform) were preserved in the final model.

### 2.3 Construction of the ensemble model

Ensemble modeling of species distribution was performed based on species presence and pseudo-absence data using Biomod2. During the process, 470 pseudo-presence data points were randomly generated for model simulation using the ‘random’ function. The model parameters were optimized, and 75% of the sample data were selected for the training model using the ‘biomod-tuning’ command; the remaining 25% of the data were used to validate the predictive performance of the model. Finally, the split-sample process was repeated ten times, resulting in 100 model simulations. We then used AUC (area under the curve), a threshold-independent measure, and TSS (true skill statistic), a threshold-dependent measure, to assess the prediction accuracy of the model. A single model with TSS ≥ 0.7 was retained, and the chosen models were combined to construct a model using the weighted average method (Allouche et al., 2006). Then, a threshold (cutoff) of 0/1 was used to identify suitable areas. The regions above the threshold were divided into two equal parts, one with medium suitability and the other with high suitability; the areas below the threshold were considered unsuitable. Finally, a map showing the areas with different suitabilities was generated using ArcGIS v10.4.1.

### 2.4 Niche changes

The background points under the present climatic conditions were selected based on the *L. gmelinii* distribution point and the 1-degree buffer distance. Through the distribution points and climate data under different climate backgrounds, the package ‘ecospat’ was used to determine the *L. gmelinii* niche overlap rate in the current scenario and under different future climate backgrounds, visualize the changes in the niche, and calculate the niche parameter D (observed value), which ranges from 0 to 1, indicating that the niche ranges from no stacking to full stacking, to assess the influence of climate change on the *L. gmelinii* niche (Di Cola et al., 2017).

### 2.5 Analysis of stand characteristics

In this study, 75 *L. gmelinii* plots (30 m × 30 m) were set up in the Greater Khingan Mountains from 2017 to 2021. Within the plot, the diameter at breast height (DBH) of trees at 5 cm height was measured, and the tree height, DBH, and plant density were investigated. The binary volume table was queried to calculate the volume per unit area, and the relative growth method was used to estimate the biomass of trees. The formula is expressed as follows (Shingleton and Frankino, 2018):


$$Y=a{\left(D^{Z}H\right)}^{b}$$

where Y is the tree biomass, D is DBH, H is the tree height, and a and b indicate the constants obtained by regression.

Two large (10 m × 10 m) shrub squares were set up at opposite corners of the plot, and three small (1 m × 1 m) herb squares were set up at the three corners of the shrub square. The R open-source package ‘spaa’ was used to determine the Shannon, Pielou, Simpson, Dma, and other indexes of the biodiversity of shrub and herb species.

Among the 75 *L. gmelinii* plots, 57 were located in the most suitable area, 14 in the marginally suitable area, and 4 in the marginally unsuitable areas. To study the possible impact of habitat suitability on stand productivity and understory plant diversity, “two-way ANOVA” in GraphPad Prism software was used to evaluate the impact of different habitat suitability values on *L. gmelinii* forest differences in subproductivity and understory plant diversity.

### 2.6 Analysis of conservation hotspots of *L. gmelinii*

The Marxan 4.0.6 model is a system protection planning model based on the simulated annealing method and is used to select the minimum cost area under certain economic constraints in a protected system (Moilanen et al., 2009; Hermoso et al., 2011). It was first used in marine protection system planning and has been widely used in terrestrial protection system planning (Munro, 2006). In the analysis, a 1 km^2^ grid was used as the research unit, and the tool “zonal statistics as table” (Spatial Analyst) in ArcGIS 10.4.1 was used to quantify the habitat distribution area of each target species in each planning unit and construct a species distribution matrix. The conservation area of *L. gmelinii* should reach 80% of the future loss of area in the potential distribution area due to climate change, and the model iterative operation was performed 100 times to obtain the optimal solution of the planning unit (Tang et al., 2021).

After the iterative calculation of the location selection, the spatial compactness of the adjusted result units was controlled by the boundary length modifier (BLM) of the model. If it is too dense, some planning units with low protection effects may be selected; as a result, the protected area distribution is too discrete (Javed et al., 2019; Tang et al., 2021). Then, the cost of the results and the relationship between the total boundary length and the total area can be analyzed *via* BLM modification. The larger the BLM value is, the more crucial the boundary cost and the smaller the fragmentation degree. Generally, the BLM value is adjusted between 1 and 10,000, and a more reasonable spatial distribution pattern of protection priority areas is obtained through repeated calculation. The final model used a model boundary correction value of 100.

## 3 Results

### 3.1 Model accuracy

All ten models ran successfully and yielded 100 (2 × 5 × 10) results. Furthermore, we optimized the model parameters using the ‘biomod_tuning’ function and verified them at each iteration according to the selected method (kappa, TSS, or ROC). The results revealed RF (Random Forest), with a kappa coefficient of 0.90, TSS of 0.98, and ROC of 0.99, as the most appropriate model for predicting the latent distribution of *L. gmelinii* (**Supplementary Figure 1**); this approach was followed by the GBM (Generalized Boosted Models), GLM (Generalized Linear Models), MARS (Multivariate Adaptive Regression Splines), and FDA (Flexible Discriminant Analysis) methods. The ANN (Artificial Neural Networks) method performed the worst among all models and failed the accuracy test. Finally, 35 best model results were chosen to construct the ensemble model (kappa coefficient of 0.92, TSS of 0.98, ROC of 1.00).

### 3.2 Current period potential distribution area

The analysis revealed (**Figure 1A**) 397,192.1 km^2^ as the current *L. gmelinii* suitable area (the sum of the marginally suitable area and the most suitable area); this region included 300,974.2 km^2^ of the most suitable area distributed mainly in the Greater Khingan Mountains in the northeastern part of the Inner Mongolia Autonomous Region and northern Heilongjiang Province. The marginally suitable area was 96,217.9 km^2^, and was distributed mainly in the grassland-forest transition zone on the east side of the most suitable area and from the Xiaoxing’an Mountains to the Zhangguangcai Mountains on the west side.

### 3.3 Predicted future potential distribution areas

**Figure 2:**
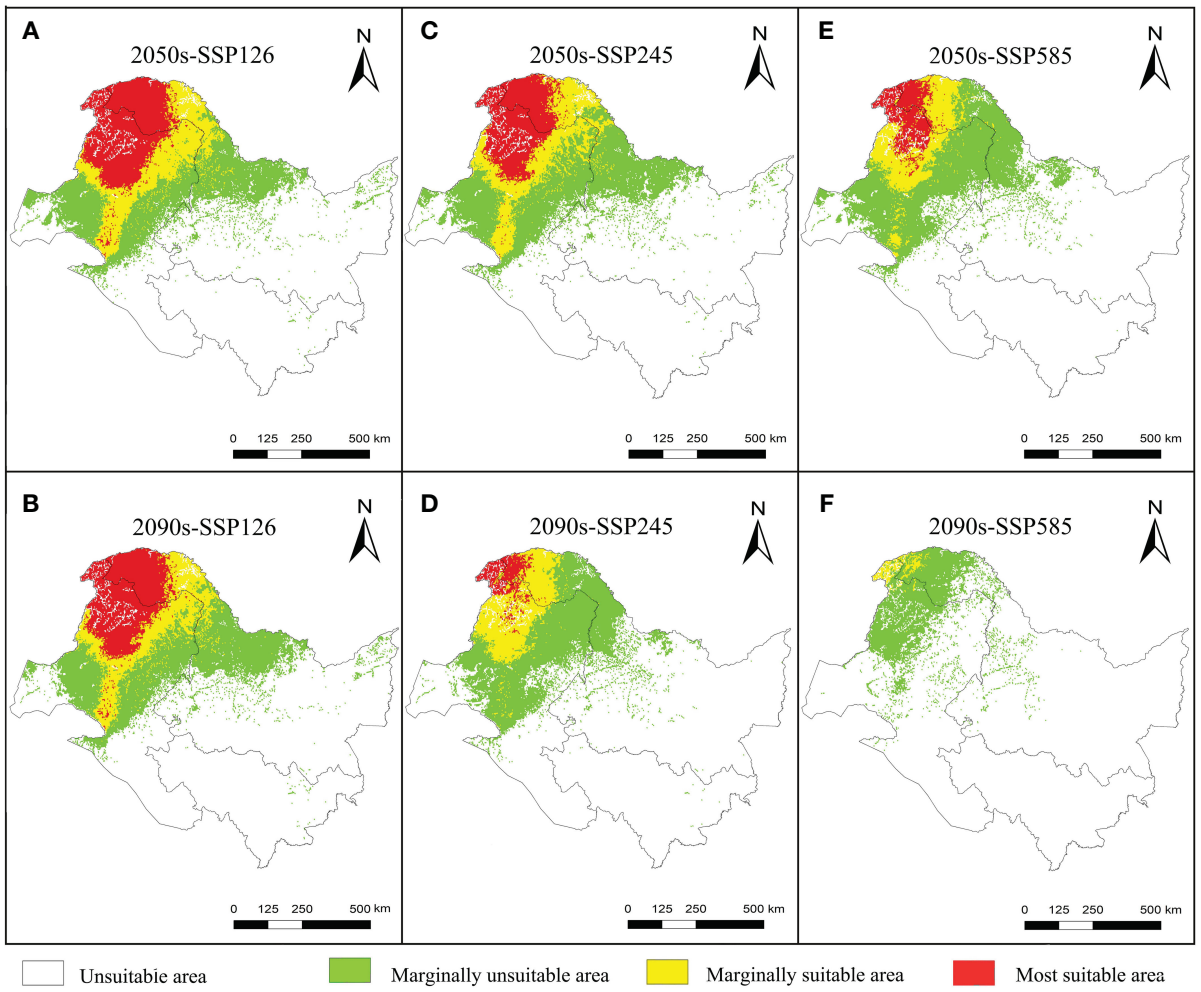
Future potential distribution of *L. gmelinii* in China.

**Figure 3:**
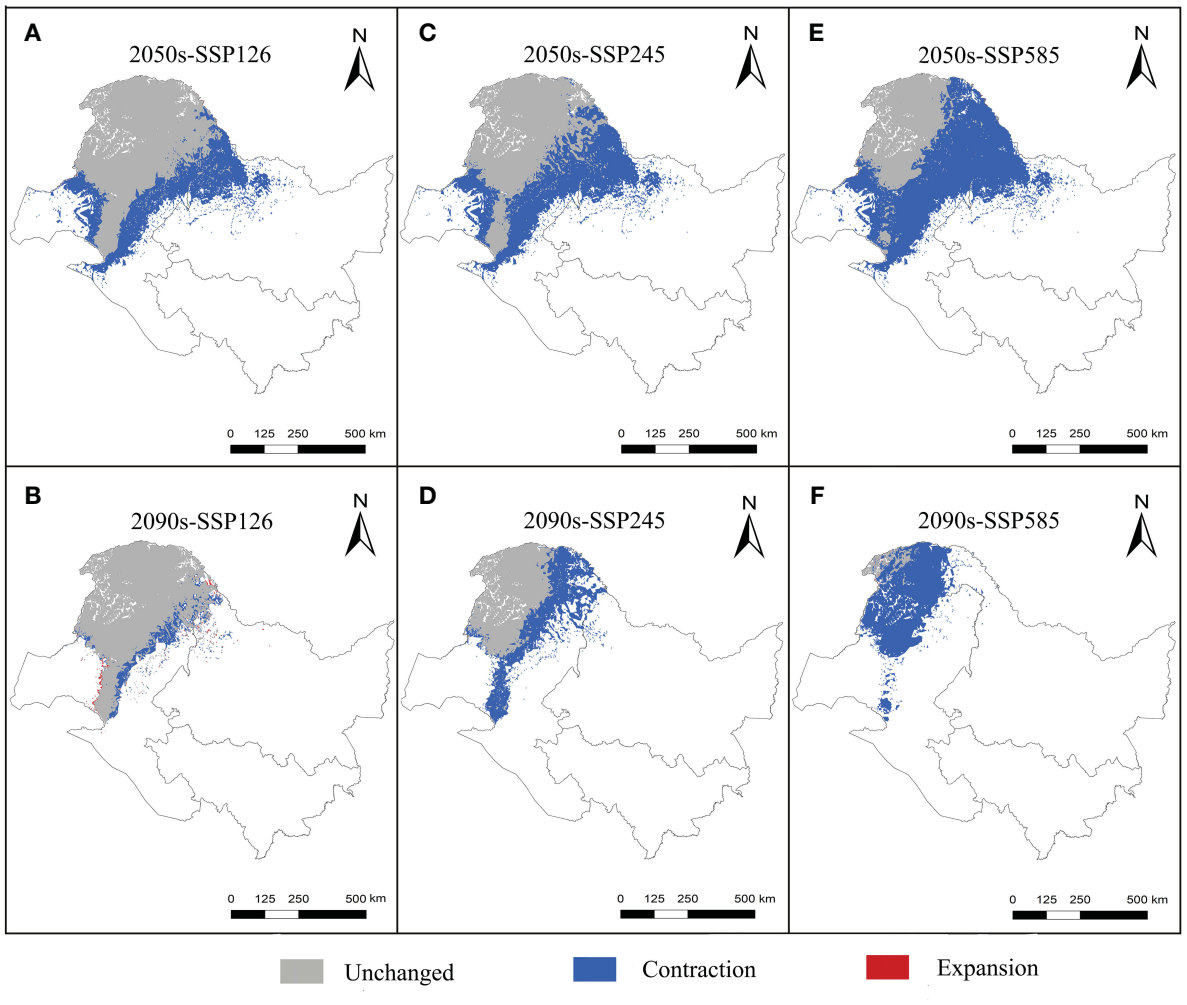
Variations in the spatial distribution pattern of *L. gmelinii* under various climate scenarios.

The suitable and most suitable areas of *L. gmelinii* will be reduced to varying degrees in the future under all climate scenarios compared with the previous period, except in the 2050s-SSP126 climate scenario, in which a small area of newly suitable areas will appear. The other climate scenarios were almost entirely contracted (**Table 1** and **Figures 2**, **3**). Under SSP126, the suitable area of *L. gmelinii* had the slightest change, and the suitable area in the 2050s is reduced by 34.8% compared with the current area, which is approximately 138 235 km^2^. The suitable area in the 2090s is reduced by 9.3% compared with that in the 2050s, which is 24,124 km^2^. Under the SSP245 climate scenario, the suitable area in the 2050s is reduced by 46.1% compared with the current total suitable area, with an area of approximately 182,985.9 km^2^. By the 2090s, the suitable area was further reduced by 43.7%, with an area of approximately 93,576 km^2^. Under the SSP585 climate scenario, the area suitable for *L. gmelinii* shrank the most, and the suitable area in the 2050s was reduced by 65.4% compared with the present suitable area, approximately 259,905 km^2^. The suitable area in the 2090s was reduced by 92.0%, with a value of approximately 126,304 km^2^, and the most suitable area will disappear entirely at this time.

**Table 1:** The spatial variations in the suitable areas for *L. gmelinii* under various climate scenarios.

Climate scenarios	Total suitable area (km^2^)	Most suitable area (km^2^)	Contraction area (km^2^)	Expansion area (km^2^)	Unchanged area (km^2^)	Contraction rate (%)	Expansion rate (%)	Unchanged rate (%)
Current	397 192.1	300 974.2	–	–	–	–	–	–
2050s-SSP126	258 956.9	137 565.8	139 339.8	84.9	258 957.0	35.1	0	65.2
2090s-SSP126	234 833.0	124 359.3	26 295.7	2 197.6	232 878.4	10.1	0.8	89.9
2050s-SSP245	214 206.2	102 728.1	183 058.4	235.0	214 036.6	46.1	0	53.9
2090s-SSP245	120 630.7	25 618.6	93 418.8	111.4	120 489.2	43.6	0	56.2
2050s-SSP585	137 287.6	48 608.1	260 226.9	120.7	137 429.1	65.5	0	34.6
2090s-SSP585	10 983.5	0	126 522.5	216.8	10 723.6	92.1	0	7.8

**Figure 4:**
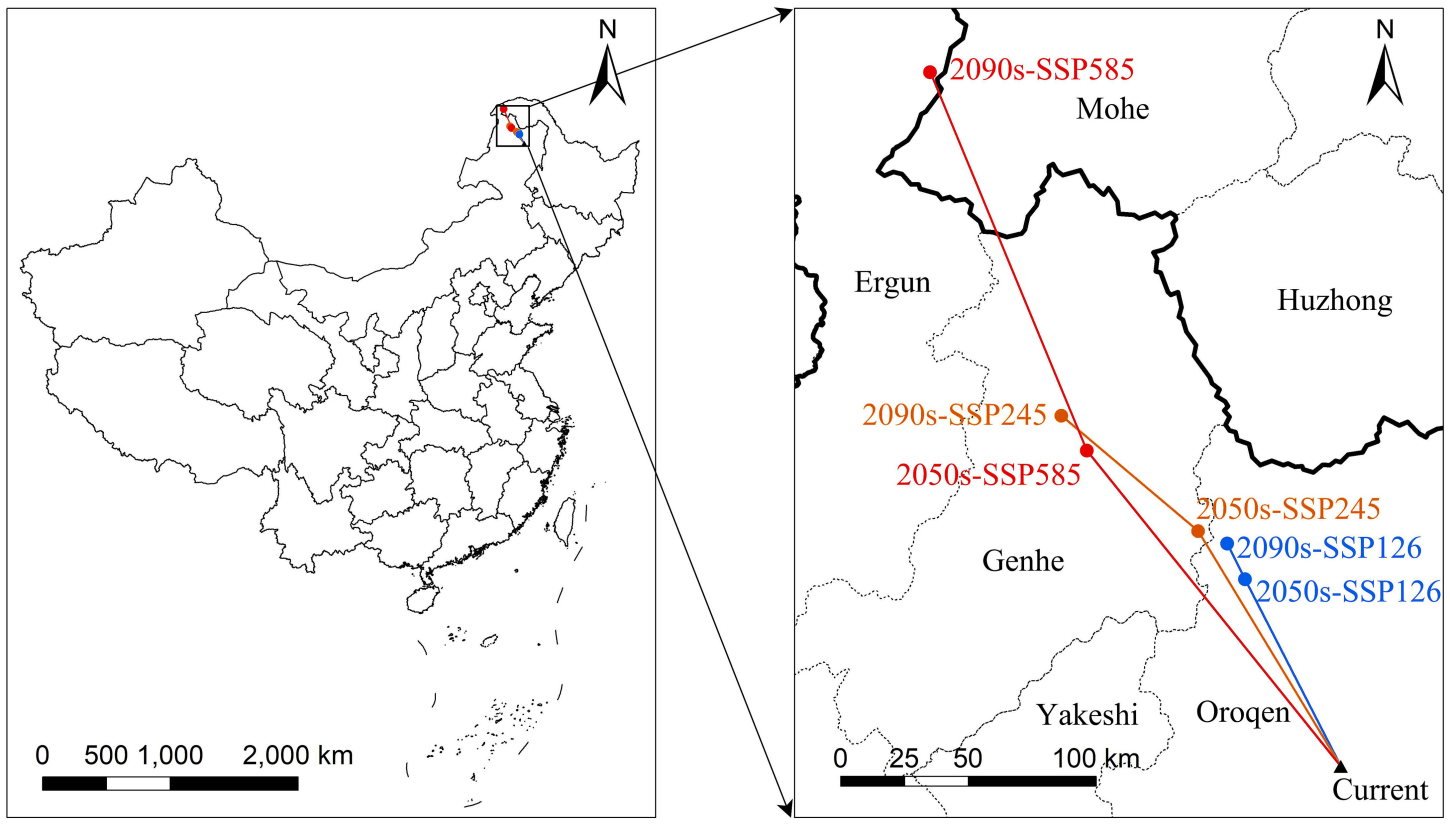
Centroid distributional shifts under different climate scenarios.

The centroids of the suitable area of *L. gmelinii* migrated northwestward in all climate scenarios (**Figure 4**). The centroid of the suitable area in the current period was located in Tozamin Township, Oroqen Autonomous Banner (123.13 E, 50.20 N). Under the SSP126 climate scenario, the centroid moved to Ganhe Township of the Oroqen Autonomous Banner (122.80 E, 50.91 N) in the 2050s, and the migration distance is 83.60 km; in the 2090s, the centroid moved to the northwest of Ganhe Township of Oroqen Autonomous Banner (122.74 E, 51.06 N), and the migration distance is 15.92 km. In the SSP245 climate scenario, the centroid moved to Hesi Street, Genhe City (122.60 E, 51.12 N) in the 2050s, with a migration distance of 109.31 km; in the 2090s, the centroid moved to Alongshan Town, Genhe City (121.96 E, 51.61 N), with a migration distance of 70.33 km. In the SSP585 climate scenario, the centroid moved to Jinhe Town, Genhe City (122.07 E, 51.47 N), with a migration distance of 160.43 km; the centroid moved to Moldauga Town, Erguna City (121.57 E, 52.92 N), with a migration distance of 164.55 km in the 2090s.

### 3.4 Future changes in ecological niche changes in the future period

**Figure 5:**
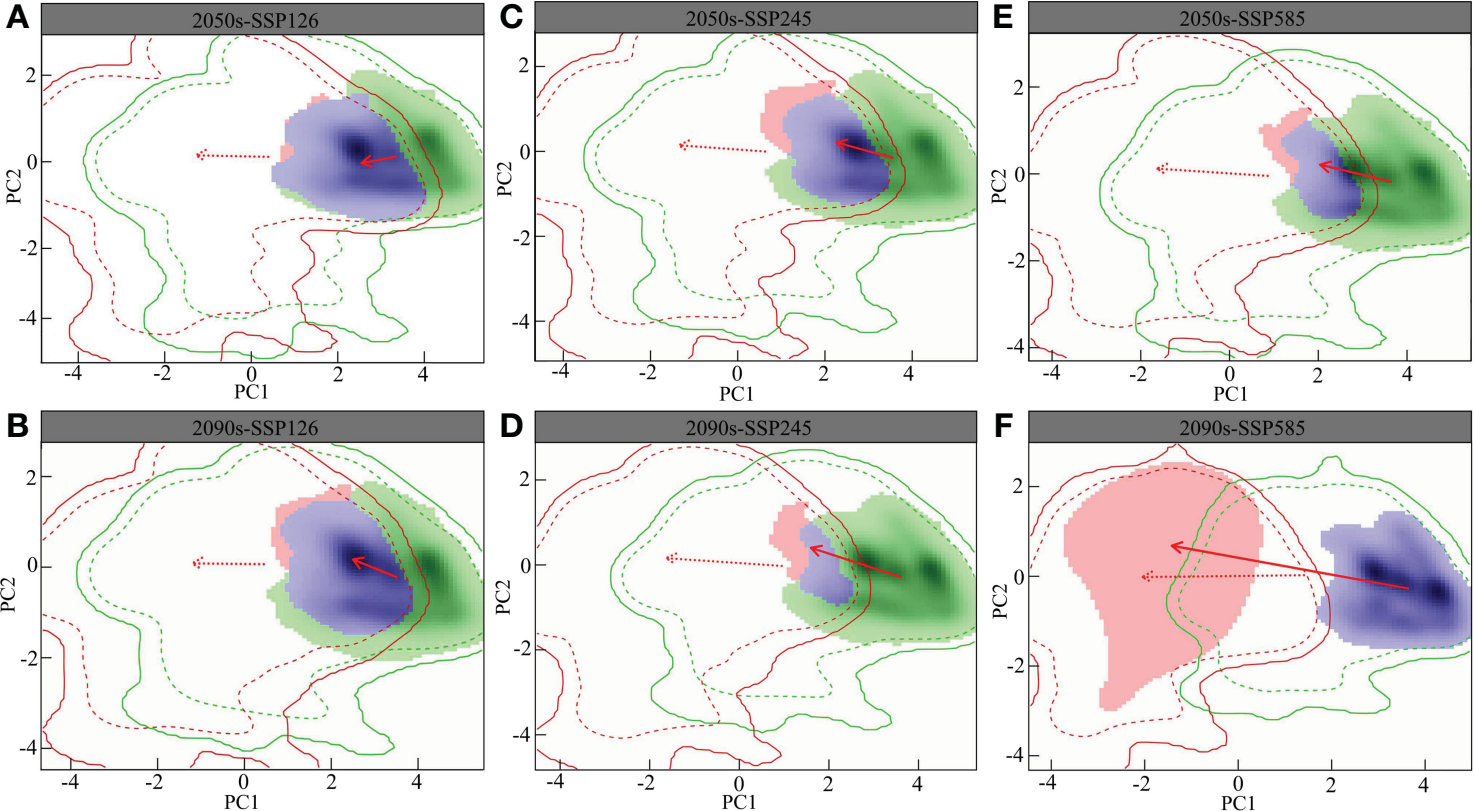
Future *L. gmelinii* niche under the SSP126 **(A, B)**, SSP245 **(C, D)** and SSP585 **(E, F)** scenarios in the 2050s **(A, C, E)** and 2070s **(B, D, F)**.

The overlap of the *L. gmelinii* ecological niche is shown in **Figure 5**. The changes in the climatic niche under different present and future climatic conditions were the same as those of the background climate. Compared with the SSP126 and SSP245 climate backgrounds, the migration distance of the climatic niche in the SSP585 climate background was longer. The *L. gmelinii* niche was completely separated from the previous period under the 2090s-SSP585 climate background. The magnitude of future global climate change considerably influences the niche shift of *L. gmelinii*, and the decreasing niche overlap indicates that *L. gmelinii* will experience a more significant niche shift during future climate change.

In the principal component analysis (PCA) of this research, the two principal components together explained 80.91%-85.15% of the variance in the environmental factors in the study area (PC1: 62.6%-70.5%; PC2: 14.65%-18.03%), i.e., the annual average temperature and the mean temperature of the coldest quarter. In addition, the average annual temperature, the mean temperature of the warmest quarter and the mean temperature of the coldest quarter were the primary factors driving the variations in the *L. gmelinii* niche, and the future climatic niche center will move closer to the mean temperature of the coldest quarter.

### 3.5 Analysis of stand characteristics under different habitat suitability values

**Figure 6:**
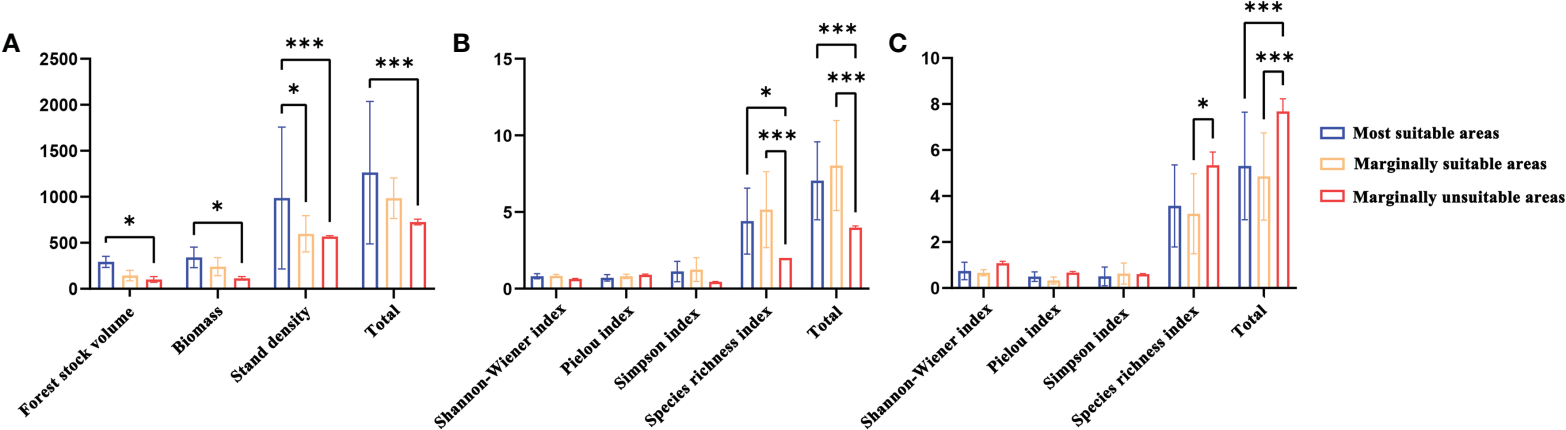
The stand characteristics **(A)** Stand productivity. **(B)** Species diversity of shrubs. **(C)** Species diversity of herbs) of *L. gmelinii* under different suitable habitats. Note: * and *** indicate significant differences at p ≤ 0.05 and p ≤ 0.01, respectively.

There were significant differences in stand productivity characteristics and understory plant diversity in the most suitable, marginally suitable, and marginally unsuitable areas (**Figure 6**). In terms of stand productivity characteristics, the stand volume, biomass, and plant density of *L. gmelinii* in the marginally suitable and marginally unsuitable areas were lower than those in the most suitable area. In terms of understory plant diversity, there were no significant differences in the Pielou, Shannon−Wiener, and Simpson indexes among the three types of suitable areas, but the *L. gmelinii* stand in the marginally suitable area had the highest understory shrub species richness index and the greatest height. The most suitable area had the second highest values, and the marginally unsuitable area had the smallest values. The *L. gmelinii* stand in the most suitable area had the highest species richness index of understory herbs, followed by that in the marginally suitable area, and the lowest value was found in the marginally unsuitable area.

### 3.6 Division of key protected areas

**Figure 7:**
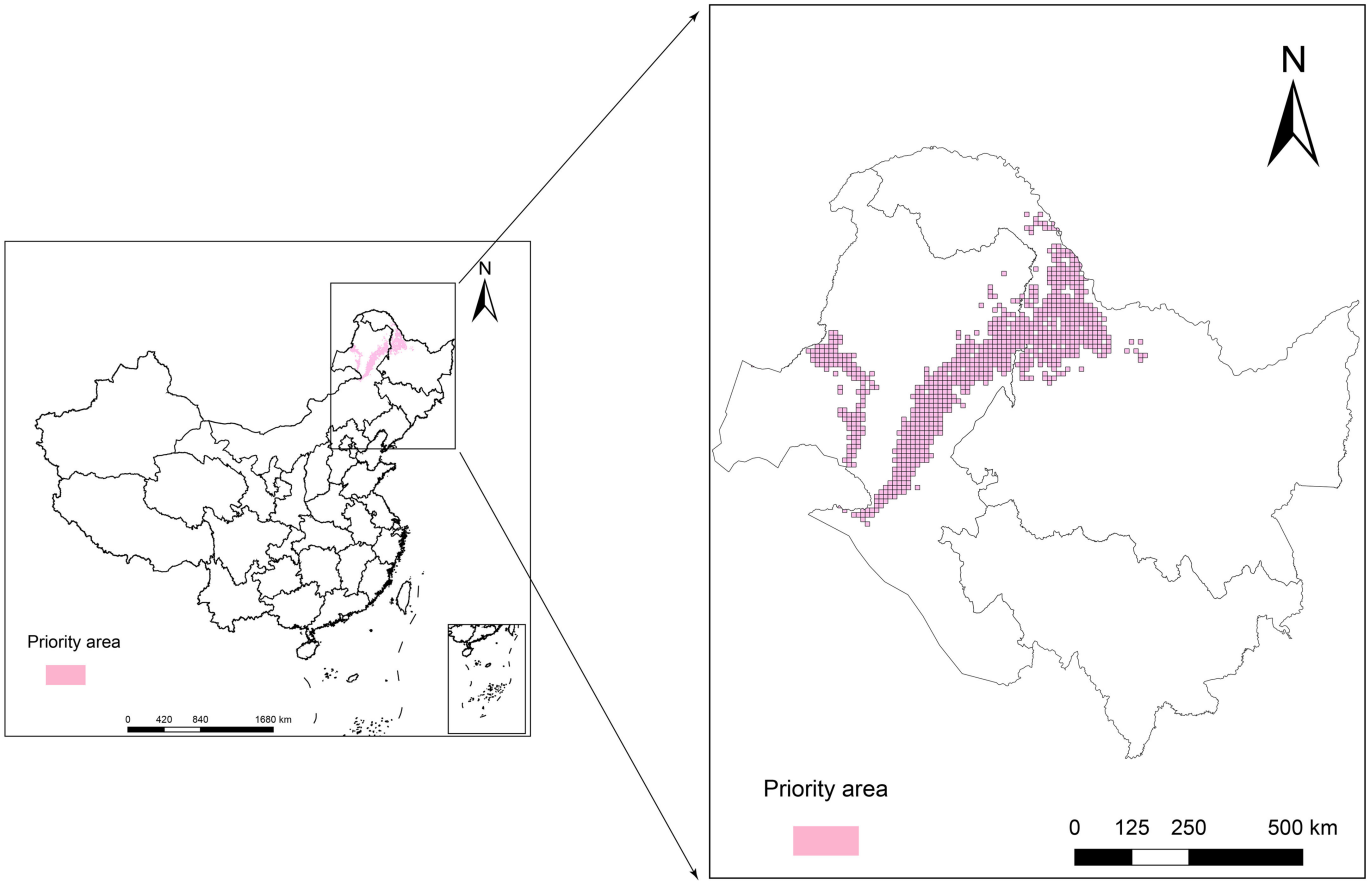
The current priority conservation areas of *L. gmelinii* in China.

**Figure 8:**
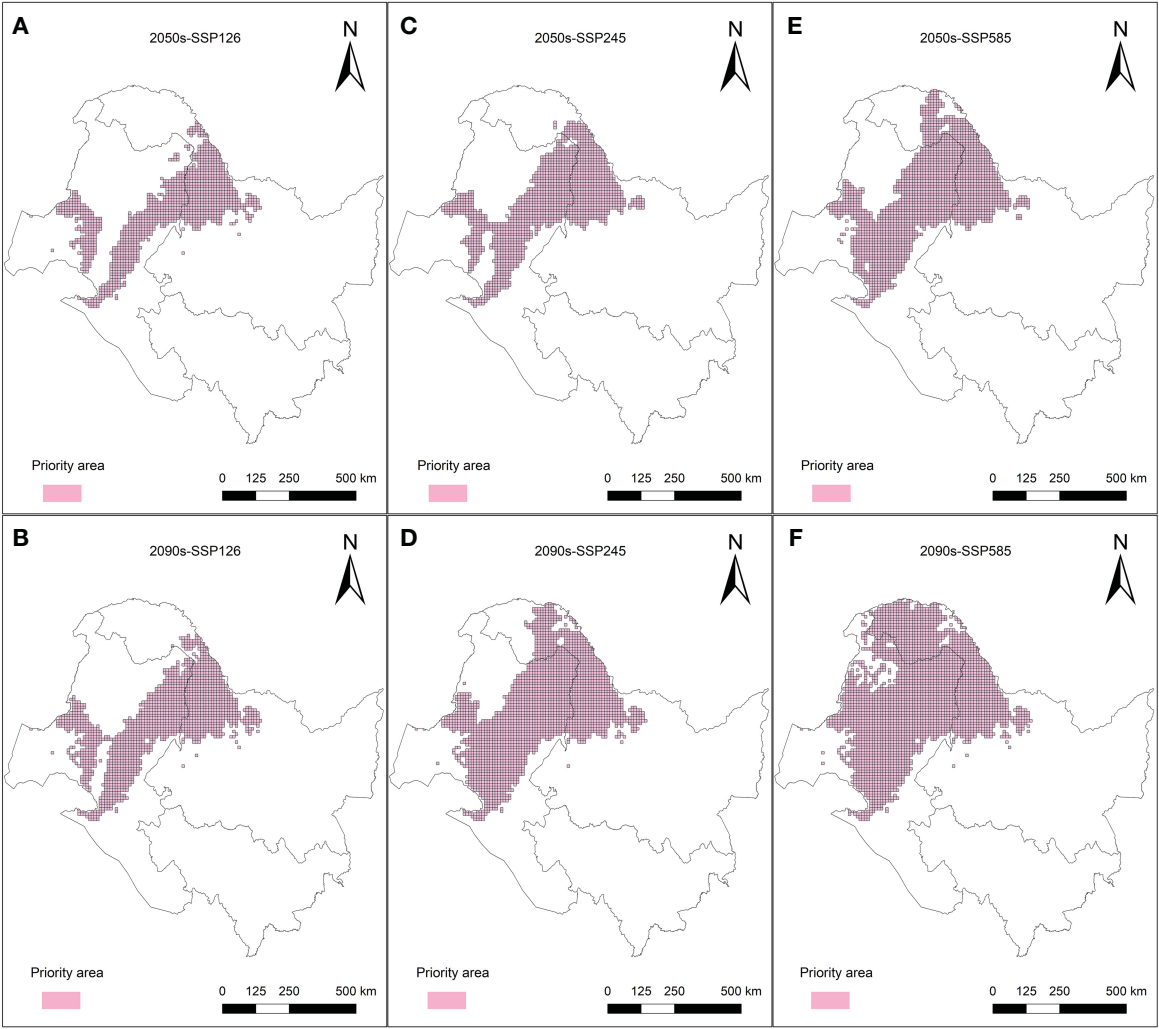
Priority conservation areas of *L. gmelinii* under the SSP126 **(A, B)**, SSP245 **(C, D)** and SSP585 **(E, F)** scenarios in the 2050s **(A, C, E)** and 2070s **(B, D, F)**.

The purpose of the protected area in the current research was to cover 80% of the area most affected by climate change in the *L. gmelinii* suitable areas under three future climate scenarios, taking into account the productivity characteristics and understory plant diversity characteristics of *L. gmelinii* in the different classes of suitable areas. The area of key protected areas in the current period was 8.38 × 10^4^ km^2^. The eastern and western sides of the Daxing’anling state-owned forests in the northeastern part of the Inner Mongolia Autonomous Region and the northwestern part of Heilongjiang Province had the main *L. gmelinii* key protected areas. The key protected areas almost completely covered the marginally suitable areas for *L. gmelinii* (**Figure 7**). In the future, the area of *L. gmelinii* priority conservation areas will be expanded together with the increase in climate change. In the 2090s-SSP585 climate scenario, the *L. gmelinii* focal area reached 28.12 × 10^4^ km^2^, and most of the *L. gmelinii* suitable areas in the current period were included in the focal area (**Figure 8**).

## 4 Discussion

With the increase in climate change challenges, the risk of *L. gmelinii* extinction in China will also increase. Currently, the potential distribution area of *L. gmelinii* is distributed in the Greater Khingan Mountains and the Xiaoxing’an Mountains. The prediction is consistent with the actual distribution of *L. gmelinii* in the temperate coniferous forests of the Greater Khingan Mountains (Fu et al., 1999). Under the climate change scenarios, the overall trend of *L. gmelinii* habitat suitability was consistent. All types of suitability migrate to higher latitudes with the intensification of climate change in the future. The rate of increase in the *L. gmelinii* suitable area was lower than the loss rate; the maximum increase rate was less than 1% of the currently suitable area. The increased area appeared only in a tiny area on the edge of the present suitable area. The areas lost were located mainly in the southeastern and southwestern parts of the present suitable areas. These areas were also the areas with the largest climatic anomalies in the results of the MESS and MoD analyses. Simultaneously, with the increase in the emission of greenhouse gases, the effect of climate change on *L. gmelinii* increased as the area of suitability change increased. Studies have shown that the continuous warming of the climate will cause temperate forest vegetation to migrate to higher latitudes. The present study’s findings agree with previous reports (Villén-Peréz et al., 2020).

The niche dynamics of *L. gmelinii* showed that the degree of niche overlap for all pairwise comparisons of current and future scenarios decreased with increasing climate change severity. The current study suggested that the annual average temperature and the mean temperatures of the coldest and warmest quarters were the main factors that caused the niche differentiation of *L. gmelinii*. Similarly, the radial growth of *L. gmelinii* was restricted primarily by temperature and was more closely related to precipitation. This result is consistent with weak growth characteristics (Zhang et al., 2018). The ecological niche of *L. gmelinii* in the SSP585-2090s climate model with the most severe climate change will be completely separated, and its original ecological niche may be occupied by other tree species moving northward. At this time, the ecosystem structure of the cold temperate coniferous forest with *L. gmelinii* as the dominant tree species will experience subversive changes, and its original biodiversity will be seriously threatened. This shift explains why the potential distribution area of *L. gmelinii* disappeared in a large area under the SSP585-2090s climate model and why the potential distribution of *L. gmelinii* continued to move northward in the future.

The productivity characteristics of *L. gmelinii* from the most suitable area were significantly better than those from the marginally unsuitable and marginally suitable areas. The most suitable area for *L. gmelinii* has a higher latitude, higher altitude and lower temperature than the marginally unsuitable and marginally suitable areas. In the Northern Hemisphere’s middle and high latitudes, the trees at the high latitude and low-temperature boundary of the tree species’ suitable area had a fast growth rate and strong adaptability, while the trees at the low latitude and high-temperature boundary of the tree species’ suitable area had a slow growth rate. (Davis and Shaw, 2001; Reich et al., 2015; Sendall et al., 2015). The growth characteristics of *L. gmelinii* from different types of suitable areas were consistent with this hypothesis. For group analysis, this study divided the *L. gmelinii* understory plant diversity into shrub species diversity and herbaceous species diversity. We found certain differences in the response of the diversity of shrub and herbaceous shrub species to changes in the habitat suitability of *L. gmelinii*. The diversity of understory shrub species in the sample plots in the marginally suitable area was significantly higher than that in the marginally unsuitable and most suitable areas, but the diversity of understory herb species in the marginally unsuitable area was slightly higher than that in the marginally suitable area. From regional to global scales, there have been related studies on plant species diversity showing significant differences due to changes in latitude and elevation, but there have still been certain differences in the conclusions drawn in these previous studies. Some studies have shown that species diversity decreases with increasing latitude and altitude (Koch, 2000), and other studies have shown that species diversity increases with increasing latitude and longitude, decreases with increasing altitude, or is higher at mid-latitudes and altitudes (Whittaker, 1960; Yang et al., 2004). The conclusions based on the analysis of *L. gmelinii* understory species diversity in this work differ from those of previous reports. The reason for this difference may be the difference in the microclimate of *L. gmelinii* forests with different habitat suitability values. Although the plant species composition of the understory has been studied, the mechanism of the influence of different stand structures of *L. gmelinii* natural forests on understory plant species diversity remains to be further studied (Zhang et al., 2017; Kumar et al., 2018).

Temperate coniferous forest is the most extensive forest ecosystem on Earth, accounting for approximately 14.5% of the land area and 30% of the forest area (Landsberg and Gower, 1997). China is located at the southernmost edge of the distribution of temperate coniferous forests in the world. The cold temperate coniferous forests are distributed only in the northern regions of the Greater Khingan Mountains in the northeast and the Altai Mountains in Xinjiang (Zhou, 1991). As the main tree species of the Greater Khingan Mountains’ temperate coniferous forest, the protection of *L. gmelinii* natural forests has been continuously strengthened, the protection system has been continuously improved, the construction of national forest parks and national wetland parks is gradually advancing, and protection efforts will increase progressively in the future (Zhang et al., 2000). However, there are still problems, such as protected areas that are too small and too scattered and fragmented in the Greater Khingan Mountains. Meanwhile, the area of spatial variation in the distribution of *L. gmelinii* is a climate change-sensitive area, which should be given attention in the conservation of *L. gmelinii*. Therefore, this study used Marxan software to assess and implement overall protection planning for *L. gmelinii* natural forests. Considering the objective situation that the residents in the Greater Khingan Mountains mainly rely on growing food crops and mining economic plants for their livelihoods, when setting the plant protection target in this study, 80% of the future loss of area in the potential distribution area was taken as the zoning target. While destroying the natural forest habitat of *L. gmelinii*, establishing protected areas should avoid serious impacts on the quality of life (QoL) and the economic development of local residents. Therefore, when setting the BLM parameters in this study, referring to the research conducted by Javed et al. (2019) and Tang et al. (2021), different BLM values and scales of planning units in the planning area will affect the final research conclusions. The BLM and planning unit areas were optimized according to the number and area of debris, and excessive encroachment on grassland and cropland in the *L. gmelinii* distribution area was avoided in the final zoning map.

## 5 Conclusions

Due to global warming, the potential distribution range of *L*. *gmelinii* will continue to decrease and move to higher latitudes, its climatic ecological niche will gradually move with it, and many areas outside the northern Greater Khingan Mountains area will become less suitable for *L*. *gmelinii*. A comparison of the characteristics of *L*. *gmelinii* stands revealed considerable differences in the productivity characteristics and understory plant diversity of *L*. *gmelinii* natural forests under different habitat suitability levels. Therefore, strengthening the conservation of cold-temperate coniferous forests in the Greater Khingan Mountains area with *L*. *gmelinii* as the main tree species and designating key protection areas based on the distribution range of highly suitable *L*. *gmelinii* habitats to reduce the damage to *L*. *gmelinii* forest resources by human activities are inevitable.

## Data availability statement

The raw data supporting the conclusions of this article will be made available by the authors, without undue reservation.

## Author contributions

Conceptualization, MG, GZ and RS. Methodology, GZ, XW and SZ. Data Analysis, MG, ZW and GZ. Writing – Original Draft, GZ, MG, LL and NT. Writing – Review & Editing, MG, GZ, SZ and RS. Supervision, RS and MG. Funding Acquisition, RS. All authors contributed to the article and approved the submitted version.
