# Supplementary material for: Priority conservation area of Larix gmelinii under climate change: application of an ensemble modeling
Source: Front Plant Sci. 2023 May 9;14:1177307. doi: 10.3389/fpls.2023.1177307 (PMC10204769; doi:10.3389/fpls.2023.1177307)
Supplement: Supplementary file 1 [file DataSheet_1.docx]

Supplementary Material

Priority Conservation Area of *Larix gmelinii* under Climate Change: Application of a Combinatorial Modeling

Minglong Gao^1†^, Guanghua Zhao^2†^, Shuning Zhang^1^, Zirui Wang^1^, Xuanye Wen^3^, Lei Liu^1^, Chen Zhang^1^, Niu Tie^4^, Rula Sa^1*^

*** Correspondence:** Rula Sa: sarula213@163.com

# Supplementary Figures and Tables

## Supplementary Figures


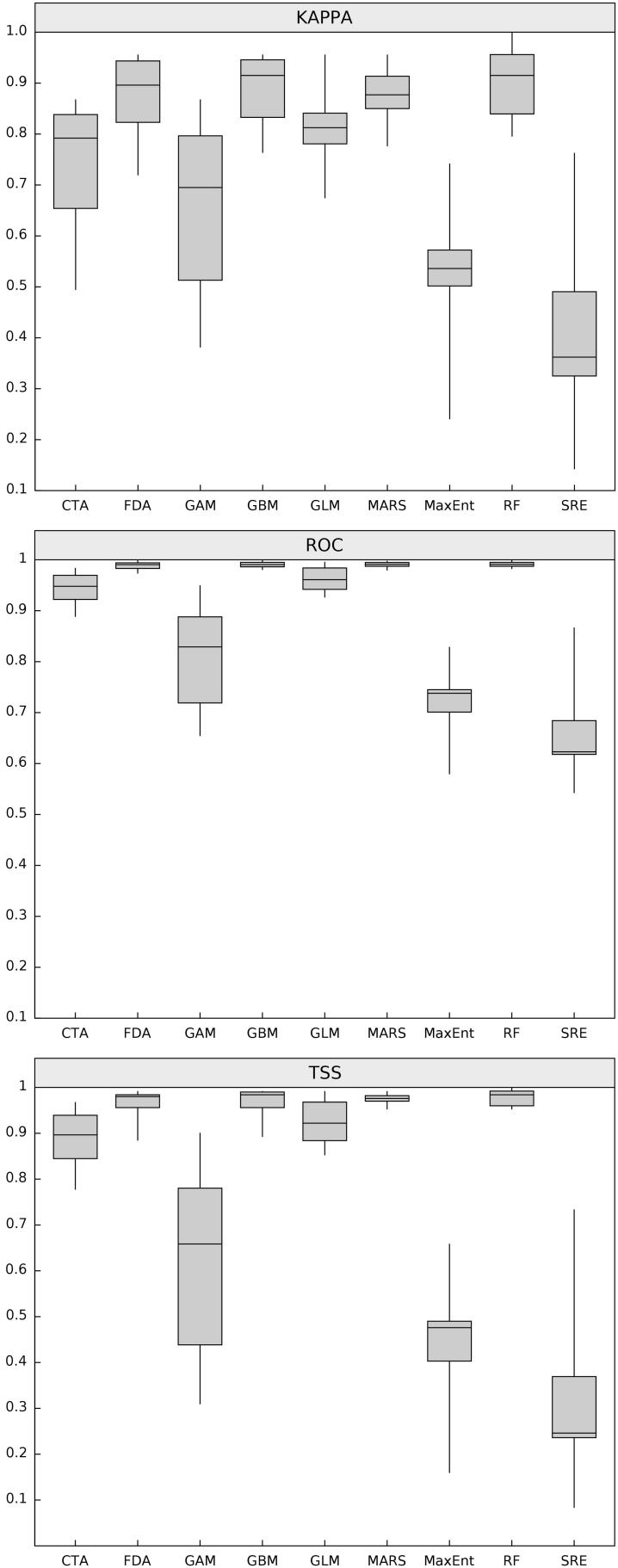


**Supplementary Figure 1.** Evaluation scores of the individual models used in the ensemble modelling.


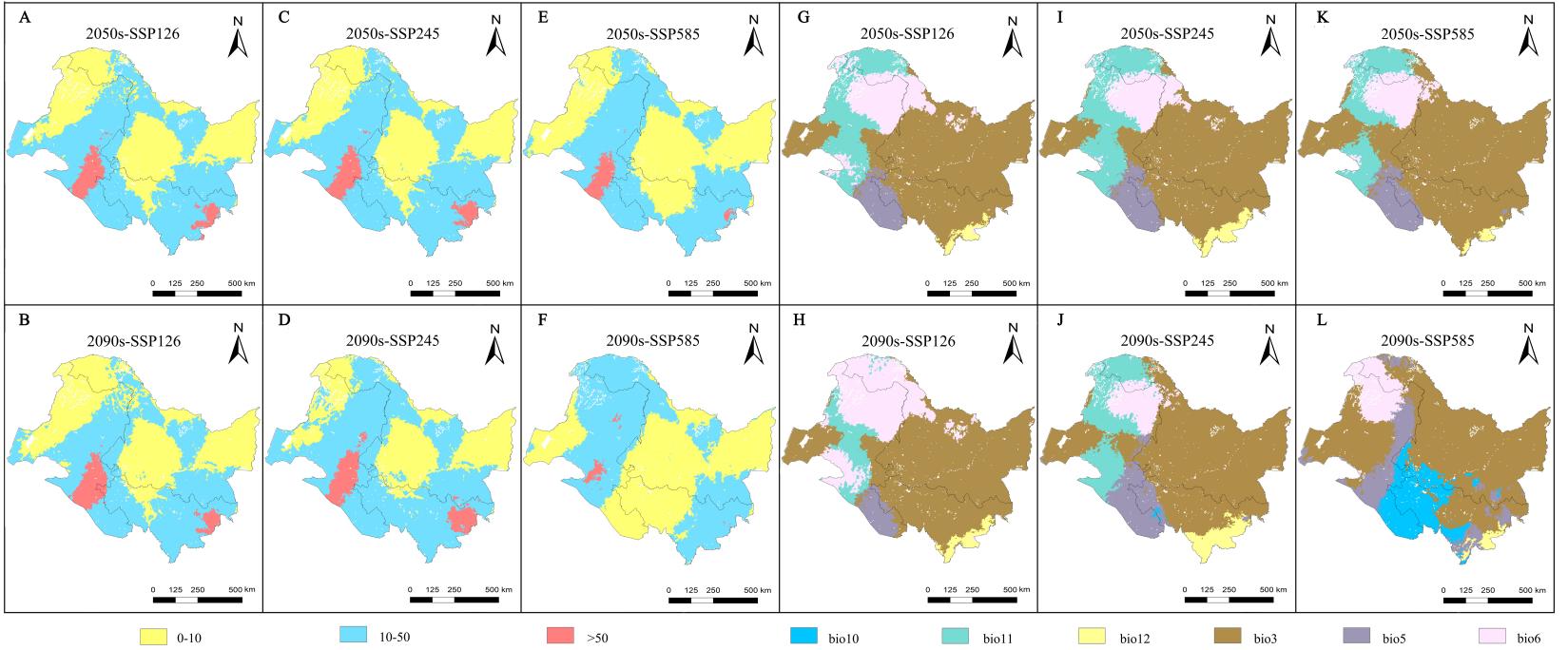


**Supplementary Figure 2.** Multivariate environmental similarity surface (MESS) and the most dissimilar (MoD) variable analysis of *Larix gmelinii* habitat distribution during different periods.


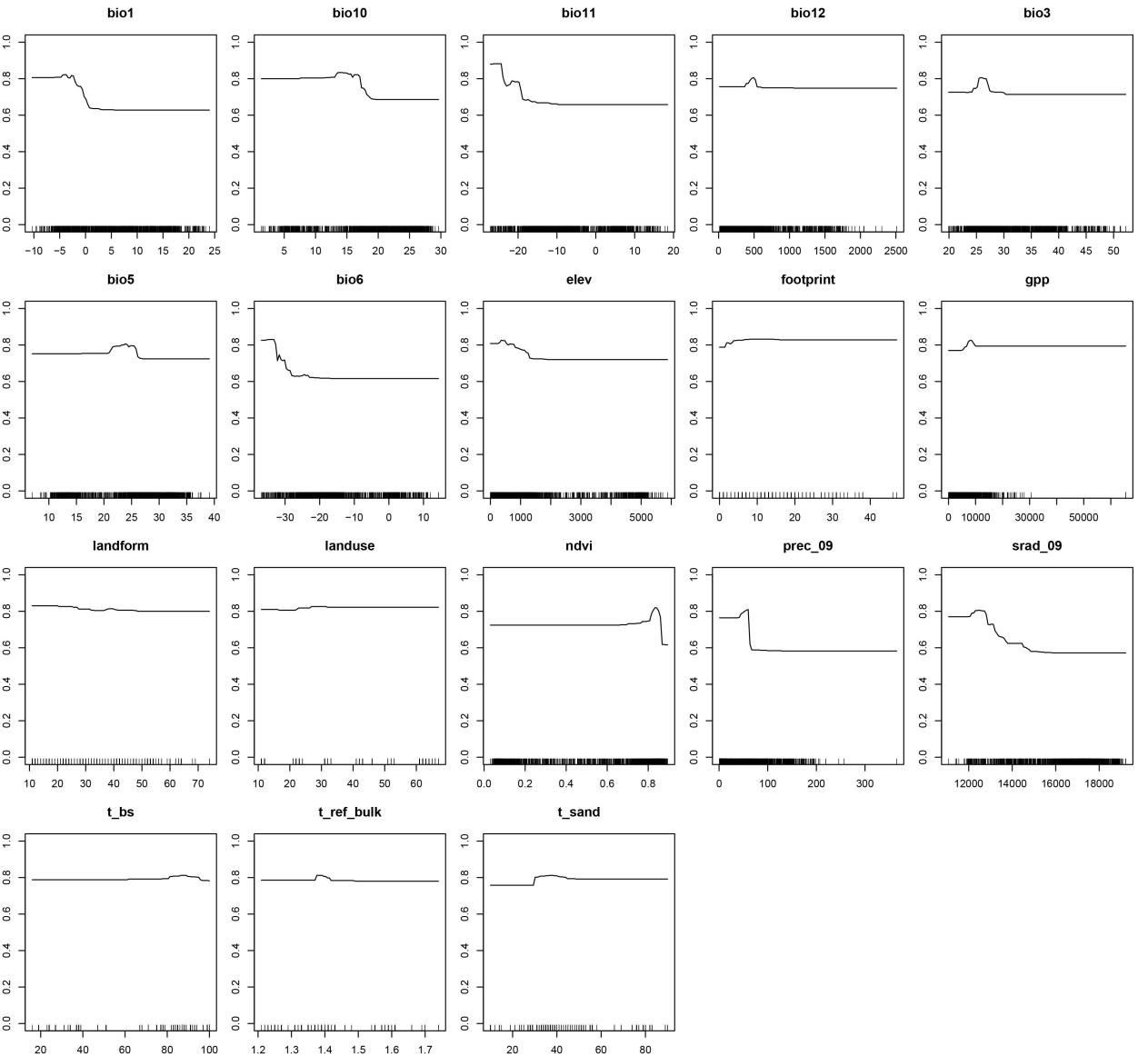


**Supplementary Figure 3.** Response curves of fourteen environmental predictors used in the ecological niche model for *Larix gmelinii*.

## Supplementary Tables

| Code | Environmental variable | Variables importance |
| --- | --- | --- |
| bio11 | Mean Temperature of Coldest Quarter | 16.4 |
| srad_09 | Solar Radiation of September | 13.9 |
| bio1 | Annual mean temperature | 12.1 |
| ndvi | Normalized Differnce Vegetation Index | 12.1 |
| bio6 | Min Temperature of Coldest Month | 11.5 |
| bio12 | Annual Precipitation | 9.1 |
| bio3 | Isothermality | 7.3 |
| gpp | Gross Primary Productivity | 5.5 |
| bio10 | Mean Temperature of Warmest Quarter | 3.6 |
| elev | Elevation | 3.6 |
| prec_09 | Precipitation of September | 1.8 |
| bio5 | Max Temperature of Warmest Month | 1.8 |
| footprint | human_footprint | 0.6 |
| landform | Land form | 0.6 |

**Supplementary Table 1.** Environmental variables and their contributions and suitable value ranges.
